# Portable Alkaline Phosphatase–Hydrogel Platform: From Enzyme Characterization to Phosphate Sensing

**DOI:** 10.3390/ijms24032672

**Published:** 2023-01-31

**Authors:** Yolanda Alacid, María José Martínez-Tomé, Rocío Esquembre, M. Antonia Herrero, C. Reyes Mateo

**Affiliations:** 1 Instituto de Investigación, Desarrollo e Innovación en Biotecnología Sanitaria de Elche (IDiBE), Universidad Miguel Hernández de Elche (UMH), 03202 Elche, Spain; 2 Instituto Regional de Investigación Científica Aplicada (IRICA), Universidad de Castilla-La Mancha, Avda. Camilo José Cela, s/n, 13071 Ciudad Real, Spain

**Keywords:** hydrogel, alkaline phosphatase, immobilization, biosensor, portable device, protein thermal stability

## Abstract

Here, we present a study on the incorporation and characterization of the enzyme alkaline phosphatase (ALP) into a three-dimensional polymeric network through a green protocol to obtain transparent hydrogels (ALP@AETA) that can be stored at room temperature and potentially used as a disposable biosensor platform for the rapid detection of ALP inhibitors. For this purpose, different strategies for the immobilization of ALP in the hydrogel were examined and the properties of the new material, compared to the hydrogel in the absence of enzyme, were studied. The conformation and stability of the immobilized enzyme were characterized by monitoring the changes in its intrinsic fluorescence as a function of temperature, in order to study the unfolding/folding process inside the hydrogel, inherently related to the enzyme activity. The results show that the immobilized enzyme retains its activity, slightly increases its thermal stability and can be stored as a xerogel at room temperature without losing its properties. A small portion of a few millimeters of ALP@AETA xerogel was sufficient to perform enzymatic activity inhibition assays, so as a proof of concept, the device was tested as a portable optical biosensor for the detection of phosphate in water with satisfactory results. Given the good stability of the ALP@AETA xerogel and the interesting applications of ALP, not only in the environmental field but also as a therapeutic enzyme, we believe that this study could be of great use for the development of new devices for sensing and protein delivery.

## 1. Introduction

Naturally occurring enzymes have been exploited, among other applications, in industry, in the manufacture of biosensors, and as therapeutic agents in the treatment of a variety of disorders and diseases [1,2,3,4,5]. In many of these applications, it is essential that the enzyme is immobilized/encapsulated to facilitate its handling and potential reuse or even to improve its stability, which is a critical step since the majority of enzymes are considered very sensitive and unstable at elevated temperatures. In addition, the enzyme requires a suitable environment to be operational [6,7,8]. Although researchers have been working on this task for decades, it remains a challenge to immobilize enzymes in a medium that allows storage for long periods of time at room temperature and high biomolecule concentrations, while maintaining their activity, and that is easily transportable and versatile to suit any application.

Immobilization on a 2D surface by means of chemical or physical interaction is the prevailing technique for protein immobilization but it has the limitation of insufficient loading capacity, among others. In this regard, 3D materials such as hydrogels can incorporate higher amounts of biomolecules, thus improving the performance of the application. These porous polymeric materials, composed of a high water content and a cross-linked polymer network, provide a biocompatible microenvironment for enzymes, allowing the diffusion of substrates and inhibitors through them [9,10,11]. However, although the aqueous medium is essential in most cases for their activity, the presence of water is not desirable for long-term storage, since the enzymes eventually lose their stability by hydrolytic degradation pathways, and aqueous medium tends to be more susceptible to bacterial contamination.

Some chemically cross-linked hydrogels can afford reversible swelling–deswelling cycles in water, returning to their original shape and mass without compromising the mechanical properties [12]. This property can be very useful in the storage of enzymes since it allows the water content of the hydrogel to be removed through different drying methods until the material is transformed into an aerogel or xerogel. If the hydration layer surrounding the protein is preserved during this process, the enzyme will maintain its activity when the hydrogel is rehydrated [13]. An example of such hydrogels are acrylamide-based hydrogels, which are biocompatible, non-carcinogenic and non-toxic [14]. Despite the interest generated by these materials, there are hardly any studies in which proteins have been immobilized in this type of hydrogels. Furthermore, the objective of these works has been more directed to characterizing and optimizing the hydrogel rather than the immobilized protein, merely to evaluate its activity [15,16].

With this in mind, we proposed to immobilize an enzyme in a hydrogel that could be dried and stored at room temperature while maintaining its properties, to be used as a portable system in order to improve its performance. The enzyme selected for the study was alkaline phosphatase (ALP), a homodimeric metalloenzyme that nonspecifically catalyzes the hydrolysis of phosphoryl esters in alkaline media and whose activity has been exploited in the design of biosensors for the direct measurement of enzyme substrates or the indirect detection of inhibitory compounds [17,18,19,20,21]. ALP has been previously encapsulated in 3D matrices such as sol–gel glasses, with satisfactory results [19,20]. However, these materials, in spite of their biocompatibility and protective properties, are fragile and easily breakable, which complicates their manipulation and possible miniaturization and portability. The use of acrylamide-based hydrogels is, therefore, a very interesting alternative for the immobilization of ALP. This protein is negatively charged at pH > 7, so it could be immobilized in cationic hydrogels by electrostatic interactions, without the need for covalent bonds that could affect its active site.

Recently, a polyacrylamide-based cationic hydrogel with high transparency and superabsorbent properties was synthesized by radical polymerization of [2-(acryloyloxy)ethyl]trimethyl-ammonium chloride (AETA) and *N,N′*-methylenebis(acrylamide) (MBA) as a cross-linker [22,23]. The hydrogel remained unaltered after several swelling-deswelling cycles and was nanostructured with graphene quantum dots to test its capacity for polyaromatic molecule sensing. Given the interesting properties of this hydrogel, in the present work we have studied its ability to immobilize ALP, exploring the conformation, stability and activity of the enzyme under different immobilization strategies to obtain the best storage conditions. The new material remains stable and functional after the drying process and subsequent rehydration, demonstrating strong interactions between the enzyme and the polymeric material. Moreover, it can be stored at room temperature for at least one month, so it could be envisaged as a portable, enzymatic system with versatile applications.

As a proof of concept, the device was used as a disposable and portable colorimetric biosensor for the detection of phosphate in water, a competitive inhibitor of ALP. The determination of this analyte is essential in environmental studies since variations in its optimum concentrations are associated with water quality. Its concentration is usually determined by conventional time-consuming analytical methods which use some carcinogenic chemicals, such as molybdenum, which are harmful to health [24,25]. Therefore, biosensors represent a promising alternative for phosphate detection, and although a large number of them have been developed so far, it is still a challenge to obtain one that allows rapid in situ determinations and that is easily usable, stable and economically viable [20,24]. In this sense, the prototype biosensor developed in this work is promising as it meets many of these requirements such as a low cost, easy handling and storage, as well as the possibility of transporting it in a miniaturized form in order to perform the determination in situ.

## 2. Results and Discussion

### 2.1. Immobilization of ALP in AETA Hydrogels

**Scheme 1:**
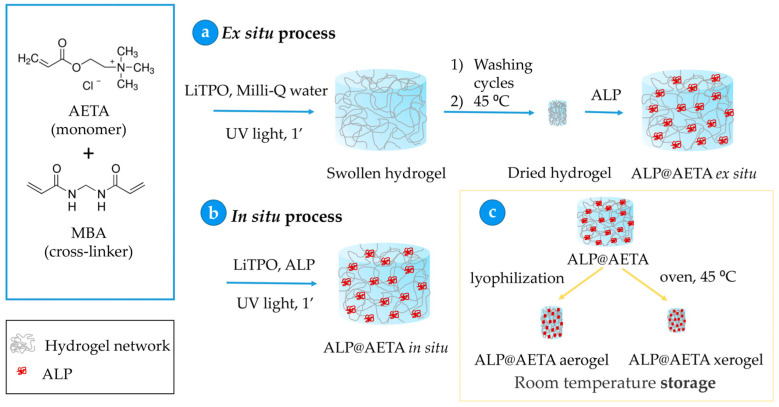
ALP entrapped process based on the incorporation of ALP (**a**) ex situ and (**b**) in situ. (**c**) ALP@AETA hydrogel storage methodology.

AETA hydrogels without and with ALP entrapped inside were fabricated as described in the Materials and Methods section (Scheme 1). The ALP-free hydrogel was generated in a few minutes under UV radiation, giving rise to a transparent matrix as shown in Figure S1a. After oven-drying for subsequent washing, the material became much more opaque and harder in consistency, making it easier to handle (Figure S1). As a first immobilization strategy (ex situ method), a weighted portion of the dried hydrogel was rehydrated in a solution containing ALP, until its complete absorption, ensuring 100% enzyme entrapment and forming the so-called ALP@AETA. As a second option (in situ method), the protein was added to the precursor, the crosslinking agent and the photoinitiator and the mixture was irradiated under the UV lamp until the ALP@AETA in situ hydrogel was formed, with the enzyme remaining entrapped inside. The hydrogel was then oven-dried at 45 °C and, after washing, a small portion was cut and rehydrated in buffer to a concentration and volume equivalent to that of the protein immobilized ex situ (approximately a cylindrical shape of 0.75 cm in diameter 1.5 cm in height). The incorporation of the protein into the hydrogel by any of the two routes did not result in visible changes in the material, which remained transparent in the hydrated state, as shown in Figure S1d, thus allowing its characterization by optical techniques.

### 2.2. Swelling Study

**Figure 1:**
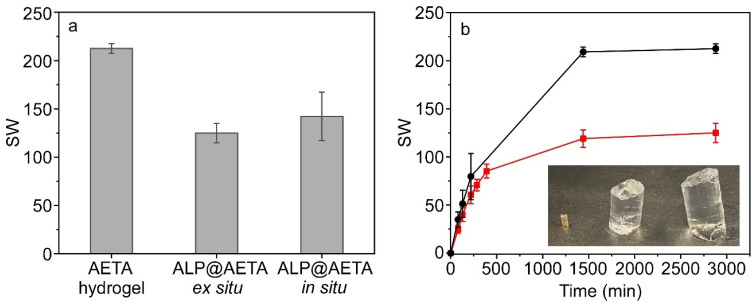
(**a**) Maximum swelling degree of AETA hydrogel, ALP@AETA ex situ and ALP@AETA in situ hydrogels (20 µM of protein). (**b**) Swelling behavior of AETA (black) and ALP@AETA ex situ hydrogel (red). Inset: Digital image of the dried hydrogel (left), the fully swollen ALP@AETA ex situ (center) and AETA (right) hydrogels. Error bars stand for the standard deviation of three replicates.

The rate and swelling degree of hydrogels control the diffusion and release of compounds through the polymer network. A high degree of swelling is desired for most of their applications; however, hydrogels in a fully swollen state usually demonstrate low strength and mechanical stability which limit their use. Stability can be enhanced via the addition of reinforcing units that interact with the cross-linked chains [26]. The fact that AETA hydrogel is a cationic network and ALP is negatively charged at pH 9 (its pI is ~4.8) suggests that for both ex situ and in situ immobilization methods, the enzyme remains anchored through electrostatic interactions which could affect some of the mechanical properties of the hydrogel. In order to explore this issue, the swelling behavior of hydrogels in the absence and presence of ALP (ALP@AETA in situ and ALP@AETA ex situ) was studied. Experiments were carried out as described in the Materials and Methods section by placing the three dried hydrogels in excess Milli-Q water and allowing them to swell. Water was absorbed by the hydrogel, thus increasing its weight over time, until the equilibrium swelling weight was reached (~24 h). The results show that all three hydrogels increased greatly in volume, with respect to the dry hydrogel, demonstrating a high swelling capacity. However, the maximum swelling degree, determined from Equation (1), was clearly higher in the hydrogel without enzyme, and was very similar in the other two hydrogels, regardless of the enzyme immobilization method (Figure 1a). The swelling rate was also faster in the absence than in the presence of ALP, as shown in Figure 1b for ALP@AETA ex situ. The inset in Figure 1b also shows that the presence of the protein improves the robustness of the hydrogel, since, in general, the experiments showed a much easier breakage of the AETA hydrogel and more difficulty in sustaining itself.

It is well known that the swelling of a hydrogel is mainly induced by the electrostatic repulsion of the ionic charges of its network [27]. In addition, hydrogen bonds can be established between the carboxylic groups of the enzyme and the amide groups of the acrylamide, resulting in a compaction of the structure that reduces the movement of the polymeric chains within the hydrogel network. Therefore, these results confirm that ALP is immobilized in the hydrogel and support the fact that the enzyme is interacting electrostatically with the cationic polymeric network, dampening cation–cation repulsions and, therefore, modifying their mechanical properties. 

### 2.3. Characterization of the Protein Inside the Hydrogel

**Figure 2:**
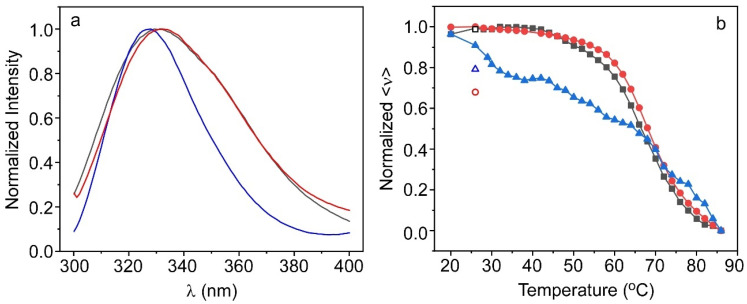
(**a**) Normalized fluorescence emission spectra recorded at room temperature (λ_x_ = 290 nm) and (**b**) evolution with temperature of the normalized mean fluorescence energy <ν> of ALP in solution (black), immobilized in situ (blue) and ex situ (red) in AETA hydrogels. <ν> values at 25 °C recorded after heat treatment are shown as empty symbols.

The intrinsic fluorescence of ALP, mainly from tryptophan residues, was used to evaluate the suitability of the immobilization processes, given the high sensitivity of tryptophan fluorescence to the polarity of the local microenvironment, and thus to the conformational state of the protein [28]. According to Bortolato et al., ALP has four tryptophan residues per monomer, mostly located in hydrophobic regions of the protein [29]. Figure 2a shows the fluorescence emission spectra of the enzyme in Tris buffer and immobilized in the hydrogel by the two methodologies (ALP@AETA in situ and ex situ). For both the free enzyme and the ex situ immobilized enzyme, the shape of the fluorescence spectrum was almost similar, with a maximum of around 330 nm, characteristic of the protein in its native state [29]. In contrast, the spectrum of the enzyme encapsulated by the in situ method exhibited a narrowing of the band, suggesting that during the polymerization process, the conformation of the protein is altered, probably due to interactions with the precursors or intermediates involved in the formation of the hydrogel, which could quench some of the most exposed tryptophans. However, the fact that no red shift of the maximum is observed suggests that the protein is not unfolded and remains in a dimeric state [30]. It should be noted that circular dichroism experiments could not be carried out as a complementary tool to characterize the conformational state of the immobilized enzymes, since the hydrogel exhibited strong absorption below 270 nm. 

An experiment was performed to determine the range of appropriate enzyme concentrations to be immobilized in the hydrogel. Figure S2 shows the area under the normalized curve of the fluorescence emission spectrum of increasing concentrations of protein in solution and ALP@AETA hydrogels formed ex situ. In both cases the behavior was similar, confirming the correct immobilization of the protein. The fluorescence intensity increased up to a protein concentration of 20 µM and above this value, it started to decrease, probably due to the effects of the inner filter and the self-absorption phenomenon [31]. Based on these results, we decided not to exceed this concentration for the rest of the experiments carried out in the work.

Changes in the fluorescence spectra of ALP were used to monitor conformational alterations of the immobilized enzyme after thermal denaturation. In the case of the ex situ immobilized enzyme, the behavior was very similar to that of free ALP, observing that, as the temperature increased, the fluorescence emission evolved towards broader, red-shifted spectra, due to the increased exposure of tryptophan to the solvent during the unfolding process. However, this effect was less pronounced for the enzyme immobilized in situ, which showed much less redshift and virtually no spectral band broadening (Figure S3).

The average energy <ν>, which selects the spectral center of mass of intrinsic protein emission after excitation at 290 nm (see Equation (2)), allows good tracking of the thermal unfolding/folding process. For ALP in buffered medium, <ν> was constant up to approximately 48–50 °C (Figure 2b). Above this temperature, it decreased cooperatively, with a denaturation temperature (T_m_) of 67.3 °C, which suggests the presence of only two structurally distinct ALP conformations, without any intermediates between native and unfolding states, in agreement with results previously reported for this enzyme [29,30]. After cooling the sample, ALP refolded to its original conformation, as reflected in the values of <ν>, which recovered its initial value before heat treatment. In the case of the ex situ immobilized protein, behavior very similar to that found in buffer was observed, with a slightly higher T_m_ value (69.2 °C), which suggests a small stabilization of the protein inside the hydrogel (Table 1). However, initial <ν> was not recovered upon cooling, evidencing that the renaturation of ALP was incomplete. The non-reversibility of the process could be due to the fact that once unfolded, new regions of the protein become exposed to the hydrogel, so additional interactions, such as electrostatic or hydrogen bonds, can be established with the polymeric network, partially hindering the protein refolding. For the in situ immobilized ALP, however, the evolution of <ν> with temperature displayed non-cooperative behavior, suggesting that the enzyme denatures through folding intermediates rather than a two-state process (Figure 2b). 

All these results support the hypothesis that the conformation of ALP is affected during the polymerization process, and that, therefore, the ex situ immobilization protocol seems to be more suitable than the in situ one.

#### 2.3.1. Enzymatic Activity Study of Ex Situ and In Situ ALP@AETA Hydrogels

**Figure 3:**
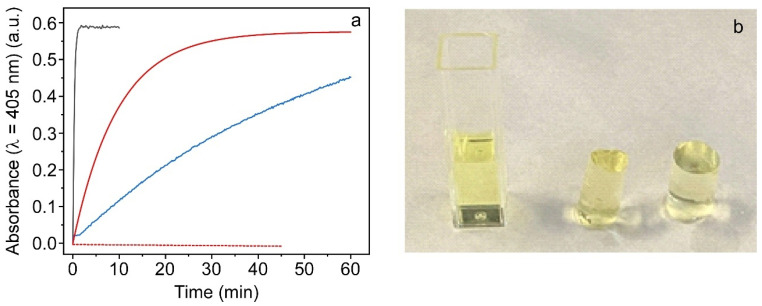
(**a**) Kinetics of PNP production by ALP in solution (black), ex situ (red) and in situ (blue) ALP@AETA hydrogels in 50 µM PNPP presence. The dashed red line represents a hydrogel in the absence of ALP to which PNPP has been added. (**b**) Comparison of the visual appearance of ALP in solution, ALP@AETA ex situ and in situ hydrogels (from left to right) after incubation with 50 µM PNPP for 20 min.

Enzyme activity was measured as described in the Materials and Methods section, monitoring the kinetics of PNP formation catalyzed by ALP (20 µM) in solution and ex situ and in situ ALP@AETA hydrogels, in the presence of PNPP (50 µM). Absorbance measurements in the hydrogel were carried out at 405 nm by placing a freshly prepared hydrogel inside the cuvette and then adding the PNPP solution, which was completely absorbed by the hydrogel. The appearance of the yellow coloration in the three hydrogels characteristic of PNP formation was indicative of enzyme activity, although the kinetics of formation differed between the three preparations (Figure 3). For ALP in solution, the absorbance of PNP reached its maximum value at ~1 min, while for ex situ ALP@AETA, the reaction was not completed until ~40 min of incubation. This slowing down of the kinetics was expected and can be attributed to diffusion restrictions imposed by the polymeric network, which limit the accessibility of PNPP to the active center of the enzyme, rather than a loss of its activity. Similar behavior has been observed for ALP immobilized in sol–gel matrices [20]. The results obtained for in situ ALP@AETA were different since, although the product was formed, the reaction was not completed even after 60 min of incubation, suggesting that the active center of most of the immobilized proteins is hardly or not at all accessible to the substrate, and/or that the conformational changes that take place during the hydrogel formation probably alter the active center shape, inactivating the immobilized enzyme. 

Taking into account both the enzymatic activity and conformational stability results, we selected the ex situ protocol to immobilize ALP in the AETA hydrogel, so in the rest of the experiments shown in this work, we will refer to the hydrogel containing the enzyme as ALP@AETA, without specifying the synthetic method. 

#### 2.3.2. ALP@AETA Stability Study 

In order to test whether the enzyme, once immobilized in the hydrogel, remained stable over time, the fluorescent properties (emission spectra and thermal behavior) of freshly prepared ALP@AETA hydrogels were analyzed for one month. For this purpose, three identical matrices were stored hydrated in the fridge at 4 °C, each of which was analyzed on different days: the first one was analyzed the day after its preparation and the other ones 21 and 30 days afterwards. The results were compared with those obtained for ALP in solution at the same time periods (Table 1). For both systems, both fluorescence spectra and thermal denaturation curves showed the same behavior during the month of storage, thus maintaining the cooperativity of the unfolding process (Figure S4).

### 2.4. ALP@AETA Storage

Storage stability of immobilized enzymes is essential for their practical applications. The results of the previous section indicate that ALP@AETA hydrogels can be stored hydrated at 4 °C for at least one month without altering either the enzyme conformation or its thermal stability. However, a hydrated hydrogel is much more unfriendly to handle than a dry hydrogel, is more difficult to transport, takes up more space and must be kept in the fridge to minimize hydrolysis processes and possible bacterial contamination. Therefore, in order to facilitate the handling and potential application of ALP@AETA, we explore the feasibility of storing the material at room temperature as a dry hydrogel. For this purpose, a long cylindrical-shaped hydrogel, as that shown in Figure S1a, was prepared and swollen with ALP. After washing, two dehydration methods were evaluated for its storage: slow drying in an air oven at 45 °C, to avoid thermal denaturation of the protein, thus obtaining an ALP@AETA xerogel; or freeze-drying to obtain an ALP@AETA aerogel. Once dry, the two materials had a solid aspect quite similar to that shown in Figure S1c, although the appearance of the aerogel was more porous and less compact than that of the xerogel. 

**Figure 4:**
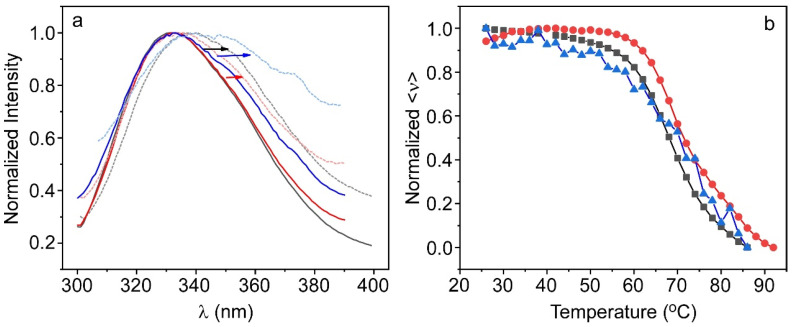
(**a**) Normalized fluorescence emission spectra (λ_x_= 290 nm) of freshly prepared ALP@AETA hydrogel (black), rehydrated ALP@AETA xerogel (red) and rehydrated ALP@AETA aerogel (blue) at room temperature (solid lines) and at 86 °C (dashed lines). (**b**) Evolution of the mean fluorescence energy <ν> of hydrated (black squares), post-drying (red circles) and post-lyophilized (blue triangles) ALP@AETA hydrogel.

ALP@AETA xerogel and ALP@AETA aerogel were kept at room temperature and, after three days, two equal-weight portions (~0.07 g) were cut from each of them, rehydrated in buffer and placed in a quartz cuvette for subsequent fluorescence analysis. Figure 4 compares the fluorescence spectra as well as the thermal denaturation curve of both preparations with those obtained for a freshly prepared ALP@AETA hydrogel, not subjected to any drying process. The fluorescence spectrum of the rehydrated ALP@AETA xerogel was similar to that recorded for the freshly prepared ALP@AETA, although upon thermal denaturation, the redshift of the spectrum was slightly higher in the rehydrated xerogel. More notable differences were observed in the emission spectrum of the rehydrated aerogel, as it red-shifted at room temperature. Moreover, the spectrum recorded after the heat treatment greatly lowered the fluorescence signal and changed considerably in shape (Figure 4a). As for the thermal denaturation curves shown in Figure 4b, the results obtained with the rehydrated aerogel showed much variability and a small loss of cooperativity, while in the case of the xerogel, the cooperativity was maintained and a slight stabilization of the enzyme was observed, obtaining a T_m_ = 72.4 ± 0.5 °C (Table 1).

These results suggest that the drying process influences the conformation of the protein, so that the oven-drying treatment preserves the conformation of ALP and even slightly increases its stability, while the freeze-drying process partially induces the denaturation of the enzyme. 

#### 2.4.1. Enzymatic Activity of ALP@AETA Aerogel and Xerogel

**Figure 5:**
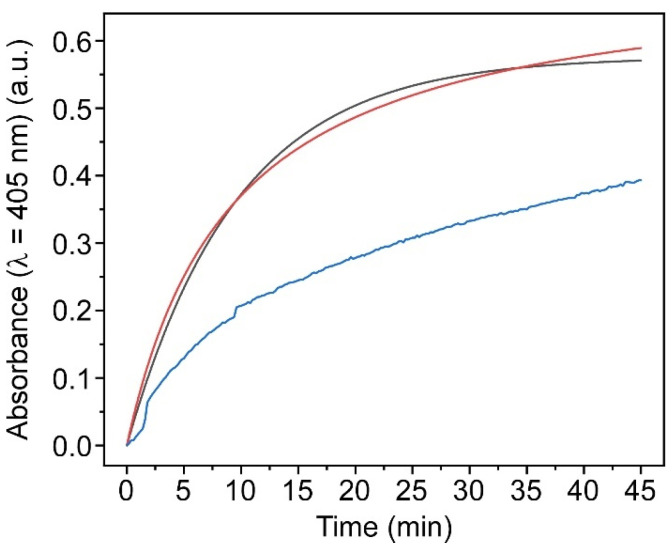
Kinetics of PNP production by alkaline phosphatase in ALP@AETA hydrogel (black), xerogel (red) and aerogel (blue) in 50 µM PNPP presence. Samples were placed in quartz cuvettes of 1 cm path length and absorbance values were collected as a function of time at 405 nm.

Thermal stability studies suggest that in contrast to expectations, it should be better to store ALP@AETA hydrogel as a xerogel rather than as an aerogel. To confirm this assumption, enzyme activity was measured in both ALP@AETA aerogel and xerogel and the results were compared with those of a freshly prepared hydrogel. As is shown in Figure 5, the kinetics of formation of PNP in ALP@AETA hydrogel and ALP@AETA xerogel showed the same pattern, reaching a plateau after ~40 min of incubation. However, the results obtained for ALP@AETA aerogel were different since, although the formation of PNP was observed, the reaction was much slower and only ~60% of substrate was transformed after 40 min. This result agrees with the fluorescence study which suggests changes in the conformation and denaturation of ALP after hydrogel freeze-drying. Moreover, the result is also in agreement with those obtained previously for ALP, which reports that, although it can be considered a freeze-tolerant enzyme, the combined freeze-drying process causes a loss of activity whose effects can be minimized by the addition of different additives, such as carbohydrates or other proteins [32]. 

In view of these results, ALP@AETA xerogel was selected as the most suitable material for storing the enzyme, discarding ALP@AETA aerogel. 

#### 2.4.2. ALP@AETA Xerogel Storage

**Figure 6:**
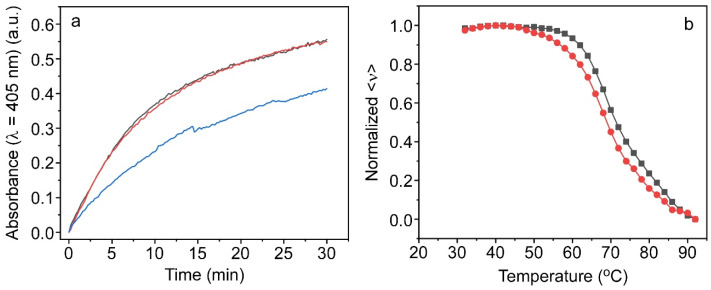
(**a**) Kinetics of PNP production by ALP@AETA freshly prepared xerogel (black), after a month (red) and after 18 months (blue) stored at room temperature. Samples were placed in quartz cuvettes of 1 cm path length and absorbance values were collected as a function of time at 405 nm. (**b**) Evolution of the mean fluorescence energy <ν> of ALP@AETA xerogels before (black squares) and after a month stored at room temperature (red circles).

As was described in the Introduction, one of the main objectives of this work is to obtain an easily manipulable material that is able to incorporate and maintain the structural and functional properties of the enzyme at room temperature, so it can be cut into small slices and taken out of the laboratory for further applications. To this end, we tested whether, after one month of storage as ALP@AETA xerogel, the enzyme remained stable and active. The study was similar to those performed previously. On the one hand, the kinetics of PNP formation was monitored and, on the other hand, the thermal denaturation curve was recorded (Figure 6). The results show that both behaviors, activity and thermal stability, were similar to those obtained for the freshly prepared xerogel, confirming the suitability of storage at room temperature. The only difference was that the recovered T_m_ value (70 °C) shifted slightly to a lower temperature but was still higher than that of ALP in buffer (Table 1). Finally, we explored whether an ALP@AETA xerogel, which had been stored in the laboratory for 18 months at room temperature, still maintained enzyme activity. The result, included in Figure 6a, shows that after this period of time, the enzyme lost about 30% of its activity, although its remaining activity allows it to be used in inhibition assays. 

### 2.5. Application: Phosphate Detection

**Figure 7:**
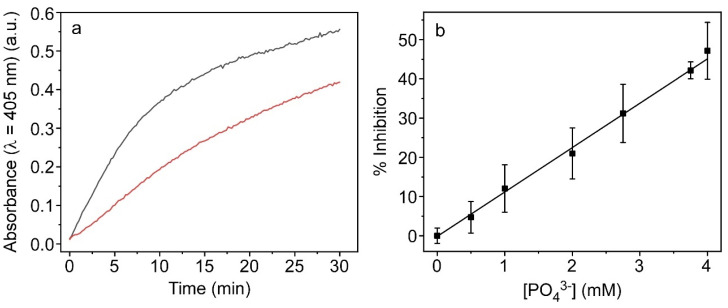
(**a**) PNP production kinetics measured in ALP@AETA after its immersion in a 50 µM PNPP solution in the absence (black line) and presence (red line) of the phosphate ion inhibitor (4 mM). Samples were placed in quartz cuvettes of 1 cm path length and absorbance values were collected as function of time at 405 nm. (**b**) % Inhibition obtained for ALP@AETA in the absence and presence of different concentrations of phosphate ion after 10 min of incubation. Error bars stand for the standard deviation of three replicates.

Once the stability of the ALP@AETA xerogel was confirmed, we considered the possibility of using the new material as a disposable colorimetric biosensor for the detection of ALP inhibitors. For this purpose, two identical slices of AETA@ALP xerogel (~0.07 g) were placed in cuvettes and both were swollen first with 0.9 mL of buffer and then with 0.9 mL of a 50 µM PNPP solution which, for one of the cuvettes, also contained 4 mM phosphate ion, a competitive inhibitor of ALP. The formation of PNP was then monitored as a function of time (Figure 7a). The results show a drastic reduction in the rate of PNP formation upon phosphate addition, indicating that AETA@ALP detects the presence of the ion and thus confirms its ability to detect ALP inhibitors. The sensitivity of the biosensor to phosphate ion was also studied. For this experiment, slices of ALP@AETA xerogels were swollen in a buffered solution containing different concentrations of phosphate ion (between 0 and 4 mM). Then, 50 µM PNPP was added and the absorbance value at 405 nm was measured in the hydrogel after 10 min incubation and plotted as % inhibition as a function of inhibitor concentration (Figure 7b). The plot was linear in the range of concentrations tested (up to 4 mM), with a limit of detection (LOD) of 511 µM, calculated from the following equation: LOD = 3σ/S, where S is the slope of the calibration curve and σ is the standard deviation of the blank. It is interesting to note that, although enzyme biosensors with a higher sensitivity to phosphate have been published in the literature (see, for example, [33,34]), one of the main advantages of the present biosensor is that it offers the possibility to perform rapid measurements of ALP inhibitors easily and at low cost outside the laboratory, using a sustainable methodology that can be extrapolated to other enzyme biosensors. 

## 3. Materials and Methods

### 3.1. Materials and Reagents

[2-(Acryloyloxy)ethyl]trimethylammonium] chloride hydrogels were synthesized by the radical polymerization of [2-(acryloyloxy)ethyl]trimethylammonium chloride solution (AETA) in the presence of *N,N’*-methylenebis(acrylamide) (MBA) as the crosslinking agent and 2,4,6-lithium trimethylbenzoylphenylphosphinate (LiTPO) as the photoinitiator, all purchased from Sigma-Aldrich (Merck Life Science, Madrid, Spain). The enzyme alkaline phosphatase (ALP) (EC 3.1.3.1; from bovine intestinal mucosa; lyophilized powder; M_w_ = 140,000 Da), the substrate p-nitrophenyl phosphate (PNPP) and the enzyme inhibitor dibasic sodium phosphate (Na_2_HPO_4_) were purchased from Sigma-Aldrich (Merck Life Science, Madrid, Spain). Stock solutions of ALP, PNPP and phosphate were prepared in Tris buffer (55 mM, pH 9) and stored at 4 °C at 45.1 µM, 2 mM and 10 mM, respectively. Tris buffer (55 mM, pH 9) was prepared by mixing Tris base and hydrochloric acid with twice-distilled and deionized water using Milli-Q equipment (Millipore, Madrid, Spain). All other solvents were of spectroscopic or analytical reagent grade (Uvasol, Merck).

### 3.2. Synthesis of [2-(Acryloyloxy)ethyl]trimethylammonium Chloride Hydrogels

Following the protocol previously described by Naranjo et al. [23], hydrogels were synthesized by photopolymerization of the cationic monomer AETA using MBA as the chemical crosslinking agent and LiTPO as the photoinitiator. In a typical experiment, 5.66 mL of AETA (26.4 mmol) and 0.01 g of MBA (0.06 mmol) were added to 5 mL of twice-distilled and deionized water. Finally, LiTPO (0.02 g, 0.06 mmol) was added to the monomer mixture. It was homogenized by gentle stirring under dark conditions. Then, 1 mL of the mixture was drawn through a disposable syringe and then exposed to UV light (λ = 335 nm) for 1 min. Finally, the tip of the syringe was cut off and the formed hydrogel of cylindrical shape was removed. The resulting material (0.3 cm diameter, 5 cm size) was immersed in twice-distilled and deionized water for 5 days, changing the water every day to remove any unreacted precursor. The washed hydrogel polymer network was then dried by heating in an oven at 45 °C for 48 h.

### 3.3. ALP Immobilization in AETA Hydrogels

ALP was immobilized in AETA hydrogels using a procedure similar to the one previously described but in two different ways: in situ and ex situ incorporation of the enzyme into the hydrogel. For most characterization experiments, the final enzyme concentration was 20 µM. After immobilization, the hydrogel was washed by water immersion and these washing water fractions were analyzed by fluorescence. In no case was protein residue found, so it is assumed that the protein was retained at 100%, regardless of the immobilization method.

- Ex situ process: Embedding ALP into the hydrogel. In this process, the previously washed and dried AETA hydrogel prepared by the above methods was immersed in ALP solution for 24 h and stored at 4 °C. This step allows loading ALP from the enzymatic solution into the hydrogel network to form ALP@AETA (Scheme 1a).
- In situ process: Incorporation of ALP into the hydrogel. Milli-Q water was replaced as solvent by 5 mL of ALP buffered solution before the polymerization process started. After irradiation with UV light, ALP@AETA hydrogels were obtained (Scheme 1b).

Finally, to facilitate handling and storage at room temperature, ALP@AETA aerogels or xerogels were prepared by removing the aqueous content by freeze-drying or by heating in an oven (45 °C, 48 h), respectively (Scheme 1c). 

### 3.4. Swelling Measurements

Swelling measurements were performed in triplicate on oven-dried hydrogels (about 0.07 g, 0.3 cm diameter, 0.6 cm size) in the absence and presence of enzyme, either using ex situ or in situ methodology. The dried hydrogels were immersed in excess Milli-Q water and stored at room temperature. The initial weight of each sample was recorded and successive measurements were taken over time as it swelled until the weight remained stable. The swelling degree (SW) was determined by applying the formula:

(1) $$\mathrm{SW}=\frac{W_{t}-W_{0}}{W_{0}}$$

where *W_t_* is the weight of the hydrogel at time *t* and *W*_0_ is the initial weight of the dry hydrogel.

### 3.5. Fluorescence Measurements

Fluorescence spectra measurements, both in solution and in hydrogel, were performed by placing the samples in 10 × 10 mm quartz cuvettes using a PTI-QuantaMaster spectrofluorometer (PTI, Birmingham, NJ, USA) interfaced with a Peltier cell. Emission spectra were acquired with an excitation wavelength of 290 nm. The fluorescence intensity of the blanks (hydrogel without protein) was checked and subtracted from the samples. For thermal denaturation experiments, heating rates of 5 °C min^−1^ and stabilization times of 30 s at each temperature were selected. Changes in the intrinsic fluorescence of ALP as a function of temperature were quantified as changes in the average energy of emission (spectral center of mass, <ν>) calculated according to Equation (2):

(2) $$<\nu >=\frac{\sum_{i}F_{i}\nu_{i}}{\sum_{i}F_{i}}$$

where F_i_ stands for fluorescence emitted at wavenumber ν_i_ [35]. The thermal denaturation temperature T_m_ of ALP was calculated by plotting the first derivative of the thermal denaturation curve. The final T_m_ value corresponds to the mean of three samples.

### 3.6. Colorimetric Activity Assay

ALP activity assays both in solution and in hydrogel were carried out in triplicate by colorimetry based on the production of p-nitrophenol (PNP), a product of the ALP-catalyzed hydrolysis reaction of PNPP, using a model UV−2700 spectrophotometer (Shimadzu, Tokyo, Japan) at room temperature. Samples were placed in quartz cuvettes of 1 cm path length. Absorbance values were collected as a function of time at 405 nm since PNP shows a strong absorption peak, maintaining a constant concentration of PNPP (50 µM).

## 4. Conclusions

In this work, we have prepared a 3D enzyme–hydrogel composite, ALP@AETA, by anchoring alkaline phosphatase ALP in a cationic crosslinked network using a green protocol. The developed material showed a high swelling capacity, resulting in a robust and transparent matrix that allowed the characterization of the immobilized enzyme by monitoring its intrinsic fluorescence. Two immobilization strategies were examined, where the ex situ methodology was found to be the most suitable since the conformational state of ALP showed minimal differences with respect to the enzyme in solution. ALP@AETA hydrogels could be stored dry at room temperature in the form of xerogels. Storage also induced additional stability of the enzyme, which slightly shifted its denaturation temperature to higher values. The xerogel could be easily manipulated and cut into small, easily transportable pieces, which were swollen with the substrate to perform colorimetric enzyme assays. As a proof of concept, the device was tested as a portable optical biosensor for the detection of phosphate ion with satisfactory results, such as the possibility to perform rapid measurements of ALP inhibitors easily and at low cost outside the laboratory, using a sustainable methodology. This methodology could be extended to the fabrication of other biosensors of interest, as well as to the storage and delivery of therapeutic enzymes.

## Figures and Tables

**Table 1:** Denaturation temperatures (T_m_) of free ALP and freshly prepared ALP@AETA hydrogels as a function of storage time at 4 °C and at room temperature for ALP@AETA xerogel. Standard deviation was determined from three trials.

	T_m_ (°C) ± SD		
t (days)	ALP Solution	ALP@AETA	ALP@AETA Xerogel
1	67.3 *±* 0.2	69.2 *±* 0.2	72.4 ± 0.5
21	67.8 *±* 0.4	69.6 *±* 0.2	
30	68.5 *±* 0.4	68.9 *±* 0.3	70.0 *±* 0.7

## Data Availability

Data reported in the study are available from the corresponding author on reasonable request.

## Supplementary Materials

The following supporting information can be downloaded at: https://www.mdpi.com/article/10.3390/ijms24032672/s1.
