# Supplementary material for: Portable Alkaline Phosphatase–Hydrogel Platform: From Enzyme Characterization to Phosphate Sensing
Source: Int J Mol Sci. 2023 Jan 31;24(3):2672. doi: 10.3390/ijms24032672 (PMC9917215; doi:10.3390/ijms24032672)
Supplement: Supplementary file 1 [file ijms-24-02672-s001.zip › ijms-2127359-supplementary.pdf]

SUPPLEMENTARY MATERIAL

Portable alkaline phosphatase-hydrogel platform: from enzyme characterization to phosphate sensing

Yolanda Alacid [1], María José Martínez-Tomé [1], Rocio Esquembre [1], Maria Antonia Herrero [2] and C. Reyes Mateo [1,*]

[1] Instituto de Investigación Desarrollo e Innovación en Biotecnología Sanitaria de Elche (IDiBE), Universidad Miguel Hernández de Elche (UMH), 03202 Elche, Alicante, Spain.
[2] Instituto Regional de Investigación Científica Aplicada (IRICA), 13071 Ciudad Real, Spain.

* Correspondence: rmateo@umh.es

KEYWORDS: Hydrogel; alkaline phosphatase; immobilization, biosensor, portable device, protein thermal stability

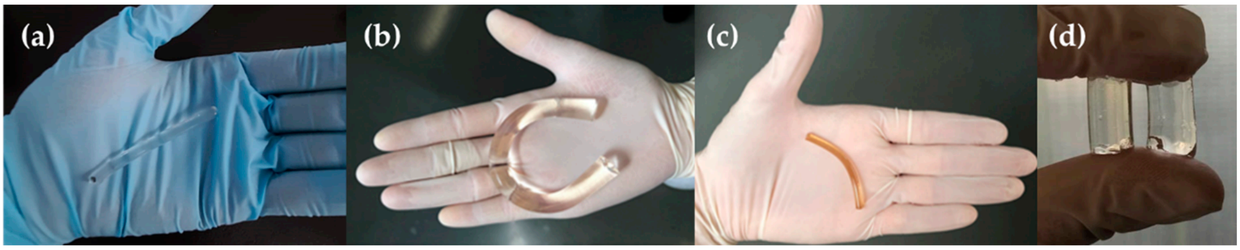

Figure S1. Digital image of **(a)** freshly prepared, **(b)** swollen, **(c)** oven-dried AETA hydrogel and **(d)** cylindrical-shaped swollen *in situ* (left) and *ex situ* (right) ALP@AETA hydrogels (0.75 cm diameter, 1.5 cm of size and 0.9 mL of solution inside).

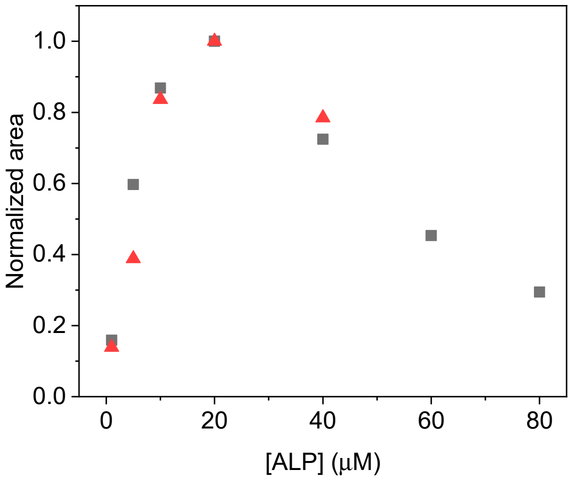

Figure S2. Area under the normalized curve of fluorescence emission spectra (λ_x= 290 nm; λ_{em}=300-400 nm) of increasing concentrations of ALP in solution (black dots) and immobilized in the hydrogel (red triangle).

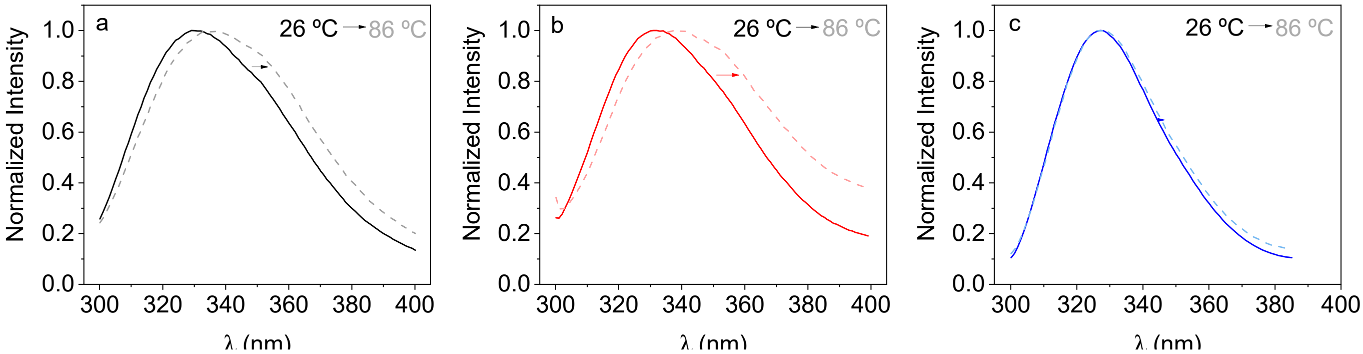

Figure S3. Normalized fluorescence emission spectra of ALP in buffered solution **(a)**, *ex situ* **(b)** and *in situ* **(c)** immobilization in hydrogel at 26 °C (solid lines) and 86 °C (dashed lines).

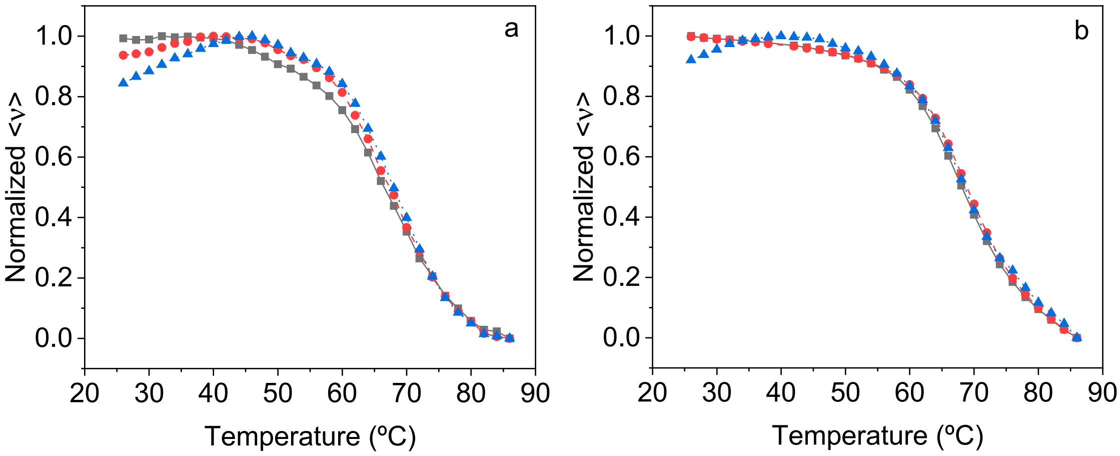

Figure S4. Evolution of the mean fluorescence energy <ν> of ALP in solution **(a)** and *ex situ* ALP@AETA **(b)** samples after 1 (■), 21 (●), 30 (▲) days stored at 4º C.
